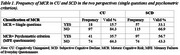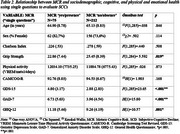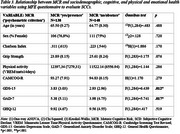# Prevalence and sociodemographic, health, emotional and cognitive profile of Motor Cognitive Risk (MCR) in the CompAS study: Comparing two procedures to evaluate cognitive complaints

**DOI:** 10.1002/alz70857_105074

**Published:** 2025-12-25

**Authors:** Fátima Fernández‐Feijoo, Lucía Pérez‐Blanco, Ana Nieto‐Vieites, Sabela C. Mallo, Sonali Arora, Maria Campos‐Magdaleno, Cristina Lojo‐Seoane, David Facal, Arturo X. Pereiro Rozas

**Affiliations:** ^1^ Departamento de Psicoloxía Evolutiva e da Educación, Universidade de Santiago de Compostela, Santiago de Compostela, Galicia, Spain; ^2^ Instituto de Psicoloxía (IPsiUS), Universidade de Santiago de Compostela, Santiago de Compostela, Galicia, Spain

## Abstract

**Background:**

Motoric Cognitive Risk (MCR) is a syndrome characterized by subjective cognitive complaints (SCCs) and slow gait speed in the absence of objective cognitive impairment and impact on activities of daily living (Verghese et al., 2012). This syndrome is strongly associated with the risk of cognitive impairment (Doi et al., 2017 & Verghese et al., 2012), but it also could be related with other factors, such as emotional components (Beauchet et al., 2021 & Xu et al., 2022). Our aims were to: 1) know the prevalence of MCR in the CompAS; 2) explore the distribution of sociodemographic, functional, emotional and cognitive status measures in MCR and non‐MCR participants; and 3) explore differences in prevalence and profiles in MCR participants using two different in two different assessment procedures.

**Method:**

An analysis of the frequencies of MCR and non‐MCR participants using two procedures to assess the SCCs was carried out in 287 pre‐symptomatic participants (i.e., CU, SCD) from the Compostela Ageing Study (CompAS). The first procedure coded the presence of SCCs if participants answered “yes” to questions on “self‐perception of cognitive worsening” and “feeling of worry about this self‐perception”. The second procedure confirmed the presence of SCCs when the participant scored greater than 6 on the MFE questionnaire (16%ile). Descriptives were calculated and differences in socio‐demographic, functional, emotional and cognitive variables were estimated using the two procedures.

**Results:**

MCR prevalence ranged from 15.7%‐33.1% and 35.7%‐56.7% respectively using the “single questions” and “psychometric criterion” (MFE questionnaire) procedures respectively (Table 1). Grip strength and emotional status were significantly worse in MCR participants when the “single questions” procedure was used (Table 2). Only emotional status was significantly worse when “psychometric criterion” was used (Table 3).

**Conclusion:**

Our results show that the “single questions” procedure is more restrictive than the “psychometric criterion” for estimating SCCs and therefore allows us to find more differences at the physical and emotional levels. Higher emotional symptomatology was observed in MCR participants regardless of the procedure used.